# CircMYOF triggers progression and facilitates glycolysis via the VEGFA/PI3K/AKT axis by absorbing miR-4739 in pancreatic ductal adenocarcinoma

**DOI:** 10.1038/s41420-021-00759-8

**Published:** 2021-11-22

**Authors:** Dandan Zheng, Xianxian Huang, Juanfei Peng, Yanyan Zhuang, Yuanhua Li, Junchi Qu, Shineng Zhang, Fengting Huang

**Affiliations:** 1 Department of Gastroenterology, Sun Yat-sen Memorial Hospital, Sun Yat-sen University, Guangzhou, 510120 China; 2 Guangdong Provincial Key Laboratory of Malignant Tumor Epigenetics and Gene Regulation, Sun Yat-Sen Memorial Hospital, Sun Yat-Sen University, Guangzhou, 510120 China; 3 Center of Digestive Endoscopy, the Eighth Affiliated Hospital, Sun Yat-sen University, Shenzhen, 518033 China; 4 Department of Gastroenterology, Tungwah Hospital of Sun Yat-sen University, Dongguan, 523000 China

**Keywords:** Pancreatic cancer, Oncogenes

## Abstract

Emerging evidence has demonstrated that circular RNAs (circRNAs) take part in the initiation and development of pancreatic ductal adenocarcinoma (PDA), a deadly neoplasm with an extremely low 5-year survival rate. Reprogrammed glucose metabolism is a key feature of tumour development, including PDA. In this research, we evaluated the role of circRNAs in reprogrammed glucose metabolism in PDA. RNA sequencing under various glucose incubation circumstances was performed. A new circMYOF was identified. Sanger sequencing and RNase R treatment confirmed its circular RNA characteristics. Real-time PCR indicated that it was highly expressed in PDA clinical specimens and cell lines. Gain-of- and loss-of-function assays showed that circMYOF induced progression in PDA. Mechanistically, RNA pull-down and luciferase reporter experiments elucidated that circMYOF, as a competing endogenous RNA for miR-4739, facilitated glycolysis via the VEGFA/PI3K/AKT pathway. Taken together, our findings indicate that circMYOF may work as a desirable biomarker and therapeutic target for PDA patients.

## Introduction

Pancreatic ductal adenocarcinoma (PDA) is a fatal malignancy with extremely high morbidity and mortality. Worldwide, the incidence of deaths caused by PDA has been gradually increasing, with a terrible 5-year survival rate [1]. Surgery offers the best therapeutic strategy for PDA patients. However, most cases take place asymptomatically, which leads to the majority of patients presenting with advanced unresectable situations. Worse, the outcomes of most chemotherapeutic agents or even immunotherapy for PDA become discouraging [2, 3]. Hence, it is beneficial to clarify the molecular mechanisms that contribute to the carcinogenesis and progression of PDA.

Reprogrammed glucose metabolism, also known as the Warburg effect, is a hallmark of malignancy [4, 5]. It exhibits increased aerobic glycolysis, glucose uptake and lactate production. It may provide rich soil for cell growth in a multicellular environment with decreased pH because of lactate production. The acidic microenvironment can brilliantly maintain their survival and enhance the migration of cancer cells [6–8]. Hexokinase (HK) induces the initial step of glycolysis, which results in the phosphorylation and localization of glucose components into intracellular organelles. HK2, one of the isoforms of HK, is a rate-limiting enzyme in aerobic glycolysis that promotes development in tumour cells [9, 10]. In addition, pyruvate kinases, especially pyruvate kinase muscle isozyme M2 (PKM2), which triggers the final and rate-determining step of glycolysis, play a crucial role in cancer progression [11–14]. The PI3K/AKT signalling pathway plays an important role in glycolysis [15, 16]. In addition, activation of the PI3K/AKT axis promotes tumour growth, invasion and metastasis [17–19]. Interestingly, it can be accommodated by diverse factors, such as hypoxia [20], HER2 [21] and vascular endothelial growth factor A (VEGFA) [22, 23].

Recent reports have illustrated that circular RNAs (circRNAs) participate in the initiation and progression of neoplasms [24–26]. Different from linear RNAs ending with 5’ caps and 3’ tails, circRNAs are characterized by the structures of covalently closed loops. Due to their conservation, tissue abundance and specificity, circRNAs may function importantly in tumour development, including PDA [27–29]. It has been reported that circFOXK2 promotes proliferation and metastasis in PDA via interactions with RNA-binding proteins (hnRNPK and YBX1) and acts as a sponge of miR-942 [30]. Interestingly, some circRNAs have the potential to encode proteins and contribute to carcinogenesis. circFNDC3B, which encodes a new protein named circFNDC3B-218aa, prohibited malignancy development and epithelial-mesenchymal transition (EMT) by accommodating snail expression in colon tumours [31].

In this research, to evaluate the biological role of circRNAs in reprogrammed glucose metabolism, we performed high-throughput sequencing and identified a novel circRNA, circMYOF (circBase ID: has_circ_0005392), which was derived from exons of myoferlin (MYOF). CircMYOF is overexpressed in PDA clinical specimens and cell lines. Moreover, gain-of- and loss-of-function assays indicated that circMYOF plays an oncogenic role in PDA. Intriguingly, we found that circMYOF, a competing endogenous RNA (ceRNA) for miR-4739, facilitated glycolysis via the VEGFA/PI3K/AKT pathway. Taken together, our findings indicate that circMYOF may be a new biomarker and treatment target for PDA patients.

## Results

### CircMYOF is upregulated in PDA tissues and cells

**Fig. 1:**
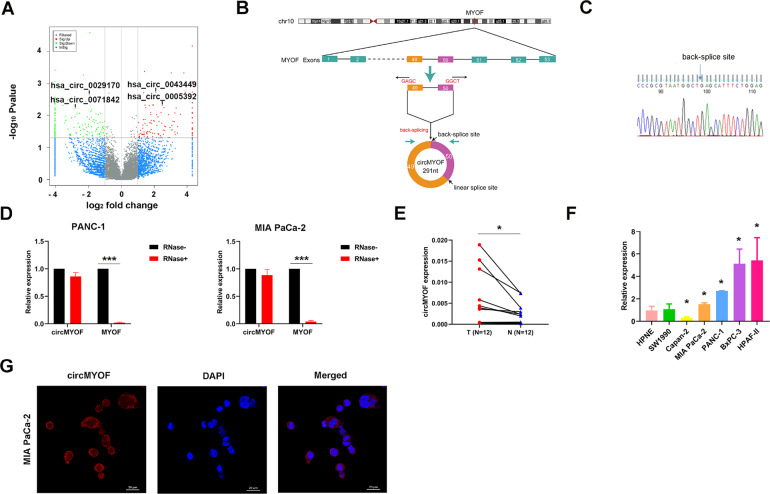
CircMYOF is upregulated in PDA tissues and cells. **A** Volcano plot analysis of differentially expressed circRNAs based on RNA sequencing in low glucose-treated and normal glucose-treated MIA PaCa-2 cells. Red plot: differentially upregulated circRNAs; green plot: differentially downregulated circRNAs. A fold change ≥2 or ≤0.5 and *p* < 0.05 were considered statistically significant. **B** Schematic illustration indicating that circMYOF was formed by the 3’ end of exon 50 and the 5’ end of exon 49 (black arrow). **C** Sanger sequencing illustrated that the back splicing site of circMYOF converged with the 3’ end of exon 50 and the 5’ end of exon 49. **D** RNase R treatment implied that circMYOF was much more resistant to RNase R treatment than linear MYOF mRNA in MIA PaCa-2 and PANC-1 cells. **E** Expression levels of circMYOF were measured in 12 PDA clinical tissues and their corresponding adjacent normal tissues by qRT-PCR. **F** The relative expression of circMYOF in PDA cell lines. CircMYOF expression was increased in most PDA cell lines. **G** RNA fluorescence in situ hybridization (FISH) was performed for circMYOF. Nuclei were stained with 4,6-diamidino-2-phenylindole (DAPI); the original magnification was 400×. Scale bars: 20 µm. Data are presented as the mean ± SD of three independent experiments. ^*^p < 0.05, ^***^p < 0.001.

Reprogrammed glucose metabolism is a key characteristic of tumour development and progression. Meanwhile, emerging evidence indicates that circRNAs play a crucial role in abnormal cancer metabolism. To deeply elucidate the biological role of circRNAs in glucose reprogramming in PDA, we first performed RNA sequencing in low glucose-treated and normal glucose-treated MIA PaCa-2 cells, which was the only one cell that can tolerate glucose-free conditions for more than 72 h among PDA cell lines (data not shown), and constructed a circRNA profiling database (GSE121596). A list of the top ten upregulated and downregulated circRNAs from RNA-seq was shown in Fig. S1. Volcano plot analysis revealed that circMYOF was one of the most overexpressed circRNAs under abnormal glucose conditions (Fig. 1A). CircMYOF is a circRNA generated from MYOF (Fig. 1B), which has not been previously investigated. Owing to its novelty, we first confirmed the circular RNA characteristics of circMYOF. Sanger sequencing was conducted to detect the back splicing site. Back splicing was converged by the 3’ end of exon 50 and the 5’ end of exon 49 (Fig. 1C). Furthermore, RNase R treatment was conducted to illustrate the stability of circMYOF. It was demonstrated that circMYOF mRNA was much more stable in RNase R treatment than MYOF mRNA (Fig. 1D). Collectively, the results indicate that the novel circMYOF obtains a circular RNA feature.

To further estimate the biological function of circMYOF, we then detected the expression of circMYOF in 12 PDA clinical samples and their corresponding adjacent normal tissues. Overexpression of circMYOF was observed in PDA tissues (Fig. 1E). In addition, circMYOF expression was enhanced in most PDA cell lines (MIA PaCa-2, HPAF-II, BxPC-3 and PANC-1) compared with that in the immortalized pancreatic cell line hTERT-HPNE (Fig. 1F). These results implied that circMYOF may contribute to pancreatic carcinogenesis. Subcellular location provided clues to the molecular mechanism of circRNAs in carcinogenesis. Hence, FISH was conducted. The results showed that circMYOF was primarily localized in the cytoplasm (Fig. 1G), which revealed that circMYOF may have functions in the cytoplasm.

### CircMYOF plays an oncogenic role in PDA in vitro and in vivo

**Fig. 2:**
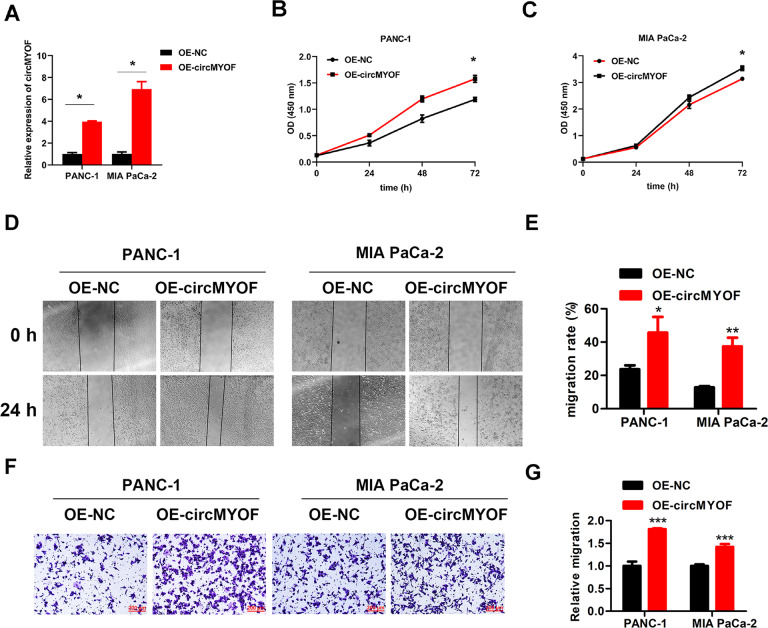
Overexpression of circMYOF promotes cell growth and metastasis in PDA in vitro. **A** CircMYOF was stably overexpressed in MIA PaCa-2 and PANC-1 cells. Effective upregulation of circMYOF was confirmed by PCR. **B, C** Cell proliferation ability was evaluated using the CCK-8 assay. Growth curve analysis illustrated that enhanced expression of circMYOF triggered proliferation in PANC-1 (**B**) and MIA PaCa-2 cell (**C**) with OE-circMYOF vectors. **D**, **E** PANC-1 and MIA PaCa-2 cells expressing OE-NC or OE-circMYOF were subjected to wound healing assays. Overexpression of circMYOF promoted metastatic potential in PDA. **F**, **G** Transwell assays indicated that circMYOF overexpression led to enhanced cell migration. Scale bars: 200 µm. Data are presented as the mean ± SD. All research was carried out triply. ^*^p < 0.05, ^**^p < 0.01, ^***^p < 0.001.

**Fig. 3:**
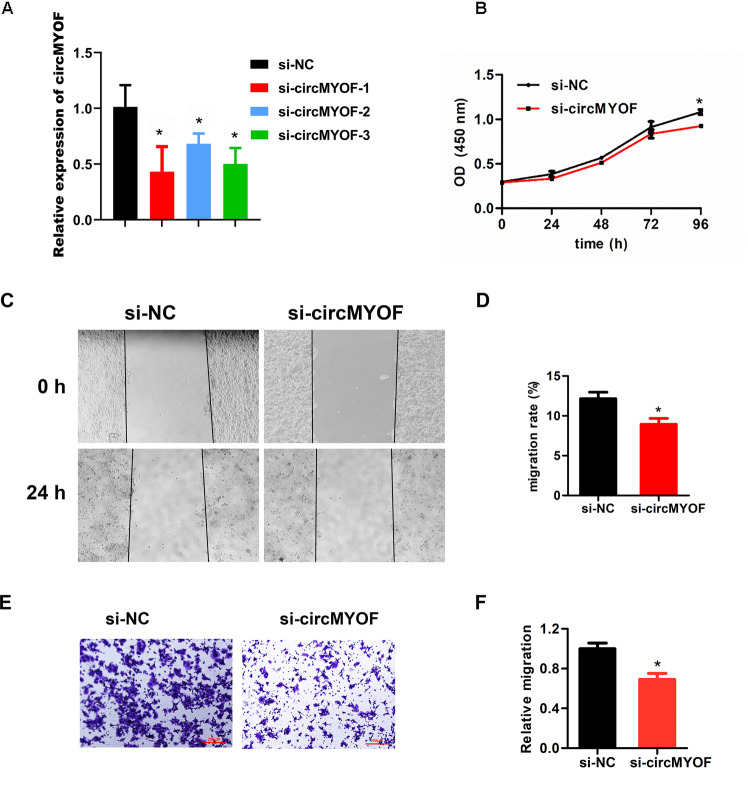
Downregulation of circMYOF prohibits cell proliferation and progression in PDA in vitro. **A** Effective suppression of circMYOF expression was performed using siRNAs in HPAF-II cells. **B** CCK-8 assay results showed that repression of circMYOF expression prohibited cell growth. **C**, **D** Wound healing experiments showed that downregulation of circMYOF expression depressed metastatic potential in PDA. **E**, **F** Transwell assay showed that attenuation of circMYOF expression inhibited migration. Scale bars: 200 µm. Data are presented as the mean ± SD of three independent experiments. ^*^p < 0.05, ^***^p < 0.001.

**Fig. 4:**
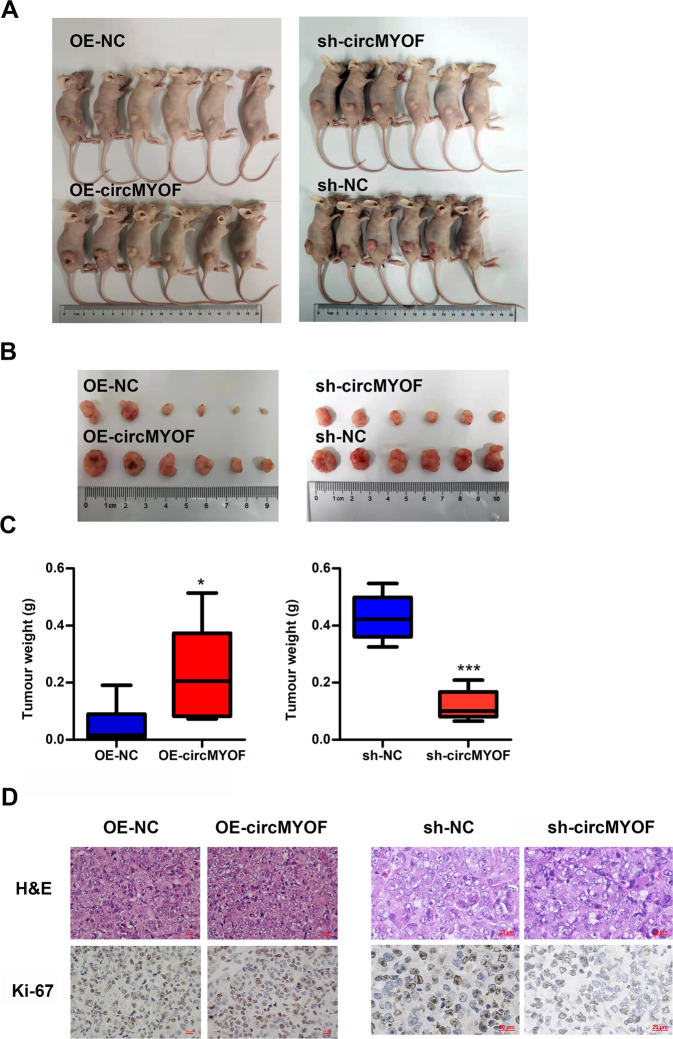
CircMYOF enhances pancreatic carcinogenesis in vivo. **A** Representative images of mouse models of upregulation (left, *n* = 6) and downregulation groups (right, *n* = 6). **B** Xenograft tumours of upregulation (left) and downregulation of circMYOF groups (right). **C** Increases of tumours weight in circMYOF upregulation (left) was observed. Decreased tumours weight was detected in circMYOF downregulation (right) group. **D** Representative images of H&E (upper) and Ki-67 (lower) staining of circMYOF upregulation (left) and downregulation (right) groups. Stronger Ki-67 staining was observed in the circMYOF overexpression group than in the control group. Weaker Ki-67 staining was observed in the circMYOF downregulation group than in the control group. Scale bars: 20 µm. Data were represented as the mean ± SD. ^*^p < 0.05.

To assess the biological role of circMYOF, gain-of- and loss-of-function assays were performed in vitro. According to the expression level of circMYOF in PDA cell lines, MIA PaCa-2 and PANC-1 cells with relatively lower expression were selected for gain-of-function assays, while HPAF-II cells with higher expression were chosen for loss-of-function assays. CircMYOF stably overexpressing cell lines were established (Fig. 2A), and no expression change of linear MYOF mRNA was observed after upregulation of circMYOF (Fig. S2A). The cell counting kit-8 (CCK-8) assay revealed that upregulation of circMYOF triggered proliferation in MIA PaCa-2 and PANC-1 cell lines (Fig. 2B, C). In addition, wound healing experiments and Transwell assays showed that overexpression of circMYOF induced metastatic potential in PDA (Fig. 2D–G). Meanwhile, effective inhibition of circMYOF expression was carried out using siRNAs in vitro (Fig. 3A), and no expression change of linear MYOF mRNA was observed after downregulation of circMYOF (Fig. S2B). Si-circMYOF-1 was chosen for further experiments. Consistent with the gain-of-function results, repression of circMYOF expression attenuated cell growth (Fig. 3B) and metastasis (Fig. 3C–F). Moreover, to validate whether circMYOF develops in vivo, xenograft models were established. As expected, enhanced growth ability was observed after upregulation of circMYOF expression (Fig. 4A–C left). Immunohistochemistry (IHC) analysis indicated stronger Ki-67 staining in the circMYOF overexpression group than in the control group (Fig. 4D left). Interestingly, decreased growth ability was observed after downregulation of circMYOF expression by sh-circMYOF-1 (Fig. 4A–C right, Fig. S3A). IHC analysis revealed weaker Ki-67 staining in the circMYOF downregulation group than in the control group (Fig. 4D right). However, no metastases were observed in the livers and lungs (Fig. S3B and S3C) maybe due to the short observation time. Taken together, these results indicated that circMYOF promotes pancreatic carcinogenesis.

### CircMYOF facilitates glycolysis via the PI3K/AKT pathway in PDA

**Fig. 5:**
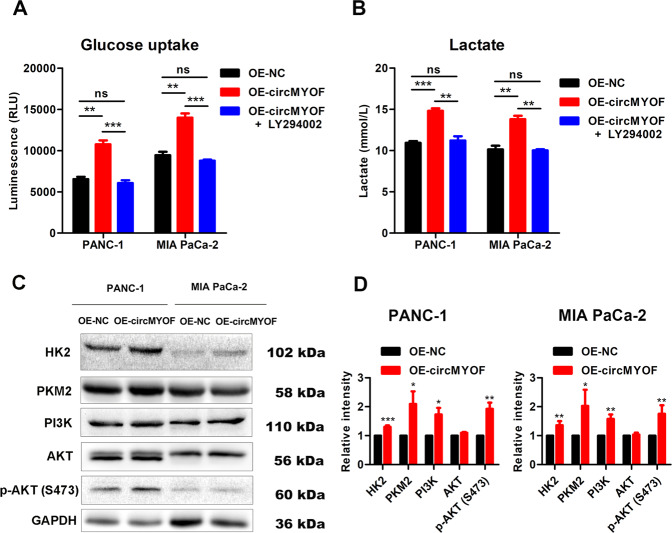
CircMYOF facilitates glycolysis via the PI3K/AKT pathway in PDA. **A, B** Overexpression of circMYOF enhanced glucose uptake (**A**) and lactate production (**B**), and LY294002 counteracted the enhancing effect of circMYOF. **C, D** Representative images of western blotting in PANC-1 and MIA PaCa-2 cells of circMYOF overexpression groups (**C**) and quantification using ImageJ software (**D**). Data were shown as the mean ± SD. All experiment was conducted in triple. ^*^p < 0.05, ^**^p < 0.01 and ^***^p < 0.001.

As shown above, circMYOF is one of the most upregulated circRNAs resulting from various glucose incubation circumstances. Hence, we wondered whether circMYOF had an effect on glucose metabolism. Glucose uptake detection and lactate production assays were conducted. Interestingly, overexpression of circMYOF enhanced glucose and lactate production (Fig. 5A, B), which indicated that an increase in circMYOF expression might be correlated with the Warburg effect. To further elucidate this hypothesis, the expression of key members of the Warburg effect, HK2 and PKM2, was detected. As expected, enhanced expression of HK2 and PKM2 was observed after overexpression of circMYOF. Considering that the PI3K/AKT pathway plays a pivotal role in glycolysis, we further explored whether circMYOF facilitates glycolysis via the PI3K/AKT pathway. Interestingly, increased expression of PI3K and phospho-AKT (Ser473) was observed after upregulation of circMYOF expression in MIA PaCa-2 and PANC-1 cells (Fig. 5C, D). Markedly, after treating OE-circMYOF cell lines with the PI3K/AKT pathway inhibitor LY294002, the enhanced glucose uptake and lactate by circMYOF overexpression were counteracted (Fig. 5A, B). These results elucidate that circMYOF facilitates glycolysis through the PI3K/AKT pathway in PDA cells.

### CircMYOF works as a sponge to miR-4739

**Fig. 6:**
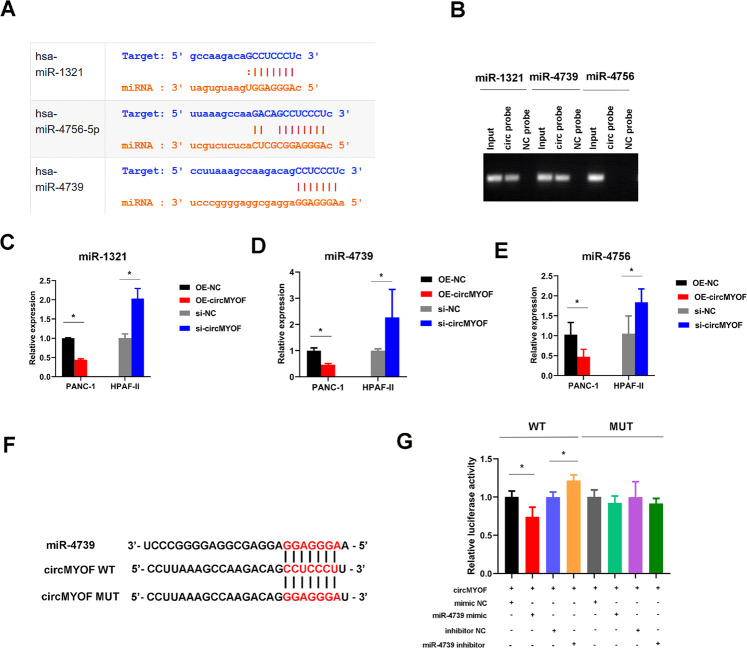
CircMYOF acts as a sponge to miR-4739. **A** Three miRNAs (miR-1321, miR-4756, and miR-4739) were selected as circMYOF downstream targets by using the StarBase v2.0 database. **B** RNA pulldown assay indicated that the biotin-labelled circMYOF probe enriched miR-4739 the most. **C**–**E** Real-time PCR demonstrated that circMYOF knockdown enhanced miR-4739 expression and that circMYOF overexpression attenuated miR-4739 expression (**D**) more obviously than miR-1321 (**C**) or miR-4756 (**E**). **F** Schematic image showing the construction of luciferase reporter vectors with the wild-type or mutant 3′ untranslated region. **G** Luciferase reporter assay illustrated that the miR-4739 mimic significantly inhibited the luciferase activity of wild-type cells, while the miR-4739 inhibitor increased the luciferase activity of wild-type cells. Data are presented as the mean ± SD. All data was evaluated in three independent experiments. ^*^p < 0.05.

Our data elucidated that circMYOF plays an oncogenic role in PDA in vitro and in vivo. However, the underlying molecular mechanism was obscure. FISH analysis implied that circMYOF was localized mainly in the cytoplasm (Fig. 1G), where the ceRNA mechanism predominately took place. The downstream miRNAs of circMYOF were predicted using the StarBase v2.0 database (Ago CLIP-seq data: strict stringency ≥5). Three miRNAs (miR-1321, miR-4756, and miR-4739) were selected (Fig. 6A). A subsequent RNA pulldown experiment showed that the biotin-labelled circMYOF probe enriched miR-4739 the most (Fig. 6B). In addition, RT-PCR illustrated that circMYOF knockdown enhanced miR-4739 expression and that circMYOF overexpression attenuated miR-4739 expression more obviously than miR-1321 or miR-4756 (Fig. 6C–E). Furthermore, to illustrate the hypothesis that circMYOF serves as a sponge for miR-4739, luciferase reporter vectors with the 3′ untranslated region of the wild-type (WT) or mutant (MUT) sequences in circMYOF were constructed (Fig. 6F). The miR-4739 mimic markedly depressed the luciferase activity of WT. Moreover, the miR-4739 inhibitor augmented the luciferase activity of WT (Fig. 6G). In summary, our data illustrate that circMYOF functions as a sponge of miR-4739.

**Fig. 7:**
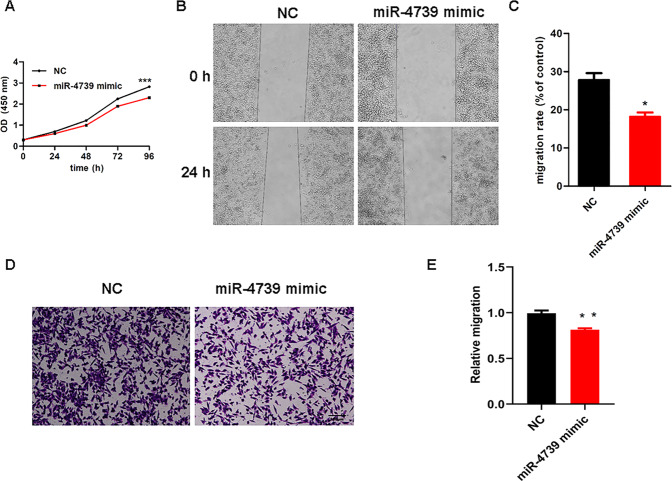
Enhanced expression of miR-4739 inhibits cell growth and development in vitro. **A** CCK-8 assay revealed that overexpression of miR-4739 depressed cell proliferation. **B, C** Wound healing assay showed that upregulation of miR-4739 expression prohibited metastasis. **D, E** Augmentation of miR-4739 expression decreased metastasis in the Transwell assay. Data are presented as the mean ± SD. All data were evaluated according to three independent experiments. ^*^p < 0.05, ^**^p < 0.01 and ^***^p < 0.001.

Additionally, we estimated the role of miR-4739 by employing CCK-8, wound healing and Transwell assays. Intriguingly, the miR-4739 mimic inhibited cell growth (Fig. 7A) and metastasis in MIA PaCa-2 cells (Fig. 7B–E).

### VEGFA is a direct target of circMYOF/miR-4739

**Fig. 8:**
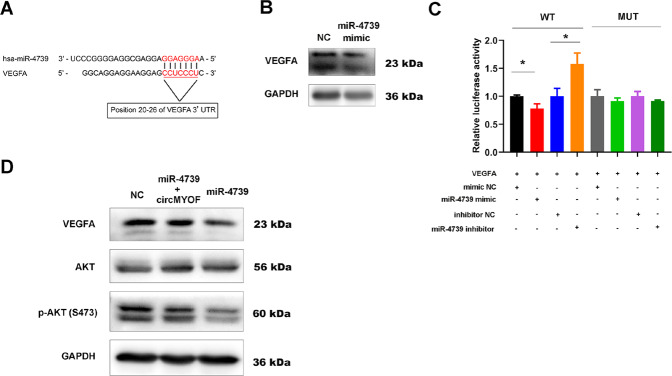
VEGFA is a direct target of circMYOF/miR-4739. **A** Schematic image showed that the potential target VEGFA obtained a similar microRNA-response element as the seed sequence of miR-4739. **B** Western blotting implied that increased expression of miR-4739 repressed VEGFA protein expression. **C** Luciferase reporter assay demonstrated that the miR-4739 mimic dramatically depressed the reporter activity of VEGFA-WT but not VEGFA-MUT. Augmentation of the reporter activity of VEGFA-WT was observed after adding the miR-4739 inhibitor (*n* = 3). **D** Cotransfection of miR-4739 mimic and circMYOF overexpression vector rescued the expression of VEGFA and inactivated the PI3K/AKT pathway. ^*^p < 0.05.

To assess the potential downstream target of miR-4739, we performed StarBase v2.0 for screening (http://starbase.sysu.edu.cn/). The potential target should obtain a similar microRNA-response element with the seed sequence of miR-4739 and circMYOF (Fig. 8A). In addition, it should be upstream of the PI3K/AKT pathway and play a vital role in activating the signalling pathway. Accordingly, we finally focused on VEGFA, which has been reported to contribute to aerobic glycolysis and carcinogenesis. Interestingly, increased expression of miR-4739 repressed VEGFA protein expression (Fig. 8B). To further elucidate this hypothesis, a subsequent luciferase reporter assay was performed. As expected, it was revealed that adding the miR-4739 mimic dramatically attenuated the reporter activity of VEGFA-WT but not VEGFA-MUT. Meanwhile, augmentation of the reporter activity of VEGFA-WT was observed after adding the miR-4739 inhibitor (Fig. 8C), which confirmed that VEGFA was a direct target of miR-4739. Intriguingly, cotransfection of the miR-4739 mimic and circMYOF overexpression vector rescued the expression of VEGFA and inactivated the PI3K/AKT pathway (Fig. 8D). Taken together, VEGFA is a target of circMYOF/miR-4739.

## Discussion

In our research, we identified a novel oncogenic circRNA, circMYOF. First, Sanger sequencing and RNase R treatment confirmed its circular RNA characteristics. Second, it is of enhanced expression in PDA tissues and cells. In addition, loss-of- and gain-of-function assays elucidated that circMYOF promotes cell growth and metastasis. Moreover, circMYOF facilitates glycolysis. Mechanistically, it acts as a miR-4739 sponge to monitor VEGFA levels and activates the PI3K/AKT signalling pathway. These data show that circMYOF plays an oncogenic role in PDA tumorigenesis.

CircRNAs, a new kind of noncoding RNA, characteristically obtain a covalently closed loop structure. Because of its stable and conserved features, it is abundantly expressed in human tissues, including cancers [32–34]. Growing evidence reveals that circRNAs play a critical role in human carcinogenesis, such as PDA. CircNFIB1 suppressed lymphangiogenesis and prohibited lymphatic metastasis by serving as a sponge of miR-486-5p in PDA [35]. CircRHOT1 promotes cell growth and development by binding miRNAs in pancreatic cancer, such as miR-26b and miR-330 [36]. In our study, circMYOF is overexpressed in PDA. Loss-of- and gain-of-function assays demonstrate that circMYOF triggers cell growth and metastatic potential in vitro. Moreover, in vivo experiments illustrated augmentation and attenuation of cell growth potential after upregulation and downregulation of circMYOF expression. These results reveal that circMYOF plays an oncogenic role in PDA. Furthermore, to explore the molecular mechanism, we first conducted FISH to identify the location of circMYOF. It was predominately localized in the cytoplasm. One of the main regulatory mechanisms of circRNAs in the cytoplasm is competing endogenous RNA (ceRNA) mechanisms [37, 38]. In summary, circRNAs function as sponges of miRNAs and subsequently modulate the targets of miRNAs, consisting of the circRNA-miRNA-protein pathway. It was reported that circACVR2A inhibited bladder cancer development via the miR-626/EYA4 axis [39]. In addition, circNRIP1 was revealed to promote development in cervical cancer via the miR-629-3p/PTP4A1 axis [40]. In our study, bioinformatics analysis and luciferase reporter experiments demonstrated that circMYOF functioned as a sponge for miR-4739. Interestingly, our further experiment demonstrated that the miR-4739 mimic inhibited cell growth and metastasis in vitro, revealing that miR-4739 plays an anti-oncogenic role in PDA.

Identification of the target of circMYOF/miR-4739 is crucial for exploring its mechanism. It is demonstrated that glycolysis plays an important role in the occurrence and development of PDA [41–43]. As proven above, circMYOF is one of the most overexpressed circRNAs resulting from various glucose incubation circumstances. It is indicated that upregulation of circMYOF expression may take part in tumour metabolic reprogramming, especially in glucose metabolism. Furthermore, it is demonstrated that circMYOF facilitates glycolysis. Therefore, we focused on key members of reprogrammed glucose metabolism, that is also known as the Warburg effect. Akt, also named “Warburg kinase”, makes great contributions to glycolysis [44, 45]. As such, the PI3K/AKT pathway is an excellent candidate [46, 47]. Interestingly, overexpression of PI3K and p-AKT was observed with no obvious change in AKT expression and upregulation of circMYOF in PANC-1 and MIA PaCa-2 cells. These results indicated that enhanced circMYOF expression activates the PI3K/AKT axis. However, PI3K was not the direct target of miR-4739 through bioinformatics analysis (data not shown). Emerging evidence demonstrates that VEGFA contributes to the activation of the PI3K/AKT signalling pathway [48, 49] and contributes to glycolysis [50, 51] and pancreatic carcinogenesis [52, 53]. Bioinformatics prediction implied that VEGFA may be the direct target of miR-4739. Attenuation of VEGFA expression occurred after enhanced miR-4739 expression. Moreover, a luciferase reporter experiment elucidated that VEGFA is the direct target of miR-4739. A rescue experiment was performed and showed that inactivation of the VEGFA/PI3K/AKT axis with upregulation of miR-4739 was recovered after cotransfection with circMYOF and the miR-4739 mimic.

At present, researches on circRNAs mainly focus on the biological function of circRNAs in diseases but seldom explore the upstream regulatory factors of circRNAs, that makes contributions to the expression of circRNAs. The factors that influence the formation of circRNAs have gradually gained attention but the researches are still superficial. RNA binding proteins (RBPs) can affect the efficiency of exon circularization and the production efficiency of circRNAs by binding to the flanking sequences of circular exons. The tissue-specific RBPs Muscleblind (MBL) of Drosophila and Quaking (QKI) of humans have been illustrated to be trans-acting factors that promote back-splicing and circRNA production [54, 55]. Meanwhile, it has been found that miRNAs, m6A modifications, and eukaryotic translation initiation factors can also play important roles in the biogenesis of circRNAs [56]. The upstream pathway on regulation of circMYOF expression is still unknown. Therefore, it is worthwhile to explore the potential regulatory factors on the expression of circMYOF in pancreatic cancer cell lines.

In summary, we identified a novel oncogenic circRNA-circMYOF in PDA. We also highlight that targeting the circMYOF/miR-4739/VEGFA/PI3K/AKT axis may be a promising strategy for the treatment of PDA. In the future, it is worthy investigating the potential regulatory mechanism on circMYOF expression.

## Materials and methods

### PDA patients and clinical tissues

Twelve pairs of PDA neoplasm specimens and adjacent nontumour clinical tissues were harvested from 2019 to 2020 at Sun Yat-sen Memorial Hospital, Sun Yat-sen University. Patients suffered no chemotherapy, immunotherapy, or radiological treatment prior to surgery. All the collected specimens were frozen immediately and kept in reserve at −80 °C. Histological diagnosis was confirmed by two pathologists. Informed consent was obtained. This research was carried out with the approval of the local ethics committee at Sun Yat-sen Memorial Hospital, Sun Yat-sen University.

### RNA sequencing

Total RNA from the PDA cell line MIA PaCa-2 cultured in low- or high-glucose medium was separated by RNAiso Plus (Takara, Japan), and ribosomal RNA was digested using a Ribo-Zero kit (Illumina, USA). Then, the linear RNA was removed using RNase R (Geneseed, China). The circRNA was broken into short pieces by reacting with the breaking reagent, and the circRNA after breaking was used as the template. One strand cDNA was synthesized with random primers, and then a two-strand cDNA was generated with a two-strand synthesis reaction system. dUTP replaced dTTP when the cDNA strand was formed, and the different joints were then connected. The UNG enzymatic method was conducted to digest a chain containing dUTP, and only one strand of cDNA with different joints of the connecting strand was retained; one strand of cDNA was purified with a kit. Then, it was repaired at the end with the A tail and connected to the sequencing connector. Then, the fragment size was selected, and PCR amplification was conducted. Afterward, the library was constructed and qualified with an Agilent 2100 Bioanalyzer (Agilent Technologies, USA). Illumina HiSeq^TM^ 2500 was chosen for sequencing. Paired-end reads were aligned to hg38.

### Cell culture and transfection

Human PDA cell lines, including MIA PaCa-2, Capan-2, SW1990, PANC-1, BxPC-3, and HPAF-II, the immortalized pancreatic cell line hTERT-HPNE and the human embryonic kidney cell line HEK-293T, were kindly acquired from the Cell Bank of the Chinese Academy of Sciences. A short tandem repeat (STR) assay was implemented to authenticate the cell lines, and mycoplasma contamination was monitored. HPAF-II was grown in MEM (Boster, USA). BxPC-3, Capan-2 and SW1990 cells proliferated in RPMI-1640 (Gibco, USA). PANC-1, MIA PaCa-2, hTERT-HPNE, and HEK-293T cells were incubated in DMEM-high glucose medium (Gibco, USA). The media above were accompanied with a concentration of 10% foetal bovine serum (FBS, Gibco, USA) in a comfortable atmosphere (at 37 °C and under 5% CO_2_).

CircMYOF sequences were inserted into the pLR-ciR vector to synthesize the circMYOF overexpression vector by Geneseed (Guangzhou, China). Stable overexpression transfection of recombinant vector was performed using polybrene (Solarbio, China) and then selected with puromycin (Solarbio, China). The siRNAs against circMYOF were constructed by Geneseed (Guangzhou, China). The siRNA sequences are listed in Supplementary Table 1. Short hairpin RNAs (shRNAs) against circMYOF were constructed by GeneChem Corporation (Shanghai, China). The sh-circMYOF sequences were listed in Supplementary Table 2. To assess miR-4739 function, a miR-4739 inhibitor and mimic were created by RiboBio (Guangzhou, China). Stable circMYOF-overexpressing PDA cells were then cotransfected with the miR-4739 inhibitor or mimic at 100 nM. Afterwards, cells were harvested for miR-4739 function detection or real-time PCR after 48 h and for western blot after 72 h of transfection. All studies were performed following the manufacturer’s instructions. PI3K inhibitor LY294002 (Cat No.: GC15485) was purchased from GlpBio (CA, USA). Cells incubated with LY294002 (10 µM) for 24 h before the following experiments.

### Extraction of RNA and real-time PCR

In brief, total RNA was isolated using RNAiso Plus (Takara, Japan) following the manufacturer’s protocol. For circRNA detection, RNase R agent was first added to decompose linear RNA, and then amplification was performed with the divergent primer. Afterwards, cDNA was generated by PrimeScript^TM^ RT Master Mix (Takara, Japan). PCR amplification was performed with TB Green^TM^ Premix Ex Taq^TM^ II (Takara, Japan) using first-strand cDNA as a template in 10 µl reaction volumes. GAPDH worked as an endogenous control. For miR-4793 detection, elimination of genomic DNA was performed first, and then cDNA was generated. The expression was normalized to U6. The 2^-ΔΔCt^ method was carried out to examine the expression level. The primer sequences are listed in Supplementary Table 3.

### RNase R assay

For RNase R detection, total RNA (2 mg) was cultured for half an hour at a suitable 37 °C with or without the use of RNase R treatment (3 U/mg) (Geneseed, China). After incubation with RNase R agents, the expression levels of circMYOF and MYOF mRNA were assessed.

### RNA fluorescence in situ hybridization (RNA-FISH)

To investigate the location of circMYOF in cells, RNA-FISH was performed using fluorescent in situ hybridization kit in accordance with the manufacturer’s protocol (Thermo Fisher Scientific, USA). Cy3-labelled circMYOF probes were procured from Geneseed. The sequence was as follows: circMYOF-Cy3: 5’-CTCCAGAAATGCTCAGCCAT-3’. Cy3-labelled circMYOF probes were incubated. Nuclei were dyed with 4′ 6-diamidino-2- phenylindole (DAPI). Finally, photos were acquired with a confocal microscope (Zeiss, Germany).

### Cell proliferation detection

Cell growth capacity was assessed with the use of Cell Counting Kit-8 (CCK-8) (APExBIO, USA). First, cells (2 × 10^3^ cells per well) were resuspended and plated in 96-well plates in 100 µl medium. Then, complete medium and CCK-8 were mixed at a proportion of 10:1, and 100 µl of the mixture was added to each well at planning time points (0, 24, 48, 72, and 96 h) after seeding. After incubation for 4 h, the relative absorbance was then examined at 450 nm with an enzyme immunoassay analyser (Thermo MK3, USA).

### Wound healing treatment

Briefly, cells were grown with complete cell culture medium in a six-well plate. When a culture of 90% confluency was reached, scratches of the cell layer were made with the tip of a 10 μl sterile pipette. Afterwards, the cells were washed with PBS. Afterwards, the culture medium was replaced with culture medium without FBS for another 24 h. Microscopic cell images of the healing wounds were collected twice at 0 and 24 h after scratching by an inverted microscope (Olympus, Japan). The numerical migration ratio data were measured by ImageJ based on the change in width in three random fields and compared to the OE-NC or si-NC group.

### Transwell assays

Cell migration detection was conducted by Transwell assay (24-well, 8 μm pore, Costar, Corning, NY, USA). Briefly, DMEM supplemented with 10% FBS (800 µl in total) was applied to the lower chamber. Cells were trypsinized, gathered, and plated in serum-free DMEM. A total of 200 μl DMEM (serum-free) with cells (4 × 10^4^) was placed into the upper chamber. Cells were cultured for 24 h at 37 °C before fixation. The non-transmembraned cells were taken away. Cells were fixed with a concentration of 4% paraformaldehyde and dyed with 1‰ crystal violet. The images were captured by an upright microscope at 100× (Olympus, Japan). The migration rate was objectively measured by calculating the number of dyed cells from five random fields by ImageJ software and comparing the results to those of the OE-NC or si-NC group.

### Western blot assay

Western blot experiment was administered as previously [57]. A total of the same amounts of proteins were added and loaded on 10–12% SDS-PAGE gels, electrically transferred to PVDF membranes, and blocked with 5% BSA. Afterwards, incubation with the membranes and various primary antibodies took place at 4 °C overnight. HK2 (Cat No.: #2867), PKM2 (Cat No.: #4053), PI3K (Cat No.: #4249), AKT (Cat No.: #9272), and p-AKT (Ser473) (Cat No.: #5012) (Cell Signalling Technology, USA) were diluted at a ratio of 1:1000. VEGFA (Cat No.: ab52917, Abcam, UK) and GAPDH (Cat No.: 10494-1-AP, Proteintech, China) were diluted at a ratio of 1:10000. Afterwards, horseradish peroxidase (HRP)-conjugated secondary antibodies were added to the membranes for blot analysis. The signal was examined with electrochemiluminescence (ECL, Millipore, Germany) using an automatic exposure imager (Syngene, UK). The relative grey value was calculated by ImageJ software.

### Animal experiment

Xenograft experiments were conducted with the approval of the Institutional Animal Care and Use Committee, Sun Yat-sen University (Guangzhou, China). Female BALB/c-nu mice (4 weeks old, 6 per group) were kindly purchased from the Laboratory Animal Center of Sun Yat-sen University. For the upregulation xenografts model, a total of 5 × 10^6^ cells per mouse were injected into the dorsal flanks. Five weeks later, the mice were sacrificed, and xenografts were collected and weighed. For the downregulation xenografts, 1 × 10^7^ cells per mouse were injected subcutaneously. Three weeks later, the mice were sacrificed, and tumours were gathered and measured. All in vivo experiments were performed following the ethical standard of the Helsinki Declaration of 1975 (1983 reversion).

### RNA pull-down assay

The biotinylated RNA pull-down experiment was implemented according to previous studies [57]. Biotinylated circMYOF probes were synthesized and labelled by a TranscriptAid T7 Kit (Thermo Fisher Scientific, USA) in accordance with the manufacturer’s instructions. Then, probe-coated beads were produced using Pierce^TM^ streptavidin magnetic beads (Thermo Fisher Scientific, USA). Cells were collected and then hatched with streptavidin magnetic beads at 4 °C for 4 h. Afterwards, the binding RNA was isolated with RNAiso Plus. RNA bands were analysed by agarose gel electrophoresis.

### Luciferase reporter experiment

The circMYOF 3′ terminal fragment or VEGFA 3’ UTR was cloned into the psiCHECK2 vector (Promega, WI, USA) and then sequenced. HEK293T cells were resuspended and cultured in a 96-well plate before transfection. After attachment, cells were transfected with miR-4793 mimic or inhibitor (RiboBio) by applying Lipofectamine 2000 (Invitrogen, USA). Forty-eight hours later, the relative luciferase absorbance was assessed by the Dual-Luciferase Reporter Assay Kit (Promega, USA). Each group of luciferase reporter experiments was evaluated in triplicate.

### Statistical analysis

All studies were conducted with three independent experiments. Continuous data are shown as the mean ± standard deviation (SD). The analysis of the data was performed with SPSS 22.0 software (SPSS, USA). For parametric data, Student’s t-test (two tails) was conducted to evaluate the data between groups. In addition, one-way analysis of variance (ANOVA) was used to examine the differences among groups. A probability of less than 0.05 was recognized as statistically significant.

## Supplementary information

Supplementary Figure Legned

Supplementary Table 1

Supplementary Table 2

Supplementary Table 3

Supplementary Figure 1

Supplementary Figure 2

Supplementary Figure 3

author contribution form

## Data Availability

The authors declare that all data supporting the findings of this study are available within the article or from the corresponding author upon reasonable request.
