# Supplementary material for: CircMYOF triggers progression and facilitates glycolysis via the VEGFA/PI3K/AKT axis by absorbing miR-4739 in pancreatic ductal adenocarcinoma
Source: Cell Death Discov. 2021 Nov 22;7:362. doi: 10.1038/s41420-021-00759-8 (PMC8608795; doi:10.1038/s41420-021-00759-8)
Supplement: Supplementary file 1 — Supplementary Figure Legned [file 41420_2021_759_MOESM1_ESM.docx]

**Fig. S1.** List of the top ten upregulation and downregulation circRNAs of RNA-seq in low glucose-treated and normal glucose incubation circumstances. CircMYOF (circBase ID: has_circ_0005392) was one of the most overexpressed circRNAs under abnormal glucose conditions.

**Fig.S2.** Effective regulation of circMYOF using suitable vectors. **A** CircMYOF stably overexpressing cell lines were established while with no expression change in linear MYOF mRNA levels. B si-circMYOF made no effect on linear MYOF mRNA expression, as measured by PCR. Data were represented as the mean ± SD.

**Fig.S3.** Effect of circMYOF on metastasis in vivo. **A** Effective inhibition of circMYOF expression using shRNAs. Sh-circMYOF-1 was chosen for the further study *in vivo*. **B** Representative images of livers and lungs of the OE-NC and OE-circMYOF groups of mice. **C** Representative photos of livers and lungs of the sh-NC and sh-circMYOF groups of mice. Data were shown as the mean ± SD. ^***^ *p* < 0.001.
