# Supplementary material for: CircMYOF triggers progression and facilitates glycolysis via the VEGFA/PI3K/AKT axis by absorbing miR-4739 in pancreatic ductal adenocarcinoma
Source: Cell Death Discov. 2021 Nov 22;7:362. doi: 10.1038/s41420-021-00759-8 (PMC8608795; doi:10.1038/s41420-021-00759-8)
Supplement: Supplementary file 2 — Supplementary Table 1 [file 41420_2021_759_MOESM2_ESM.docx]

Supplementary Table 1. Oligonucleotides sequences of si-circMYOF

| siRNA sequences | Sequence (5’-3’) |
| --- | --- |
| si-circMYOF-1 | CGCGTAATGGCTGAGCATT |
| si-circMYOF-2 | CGTAATGGCTGAGCATTTC |
| si-circMYOF-3 | GTAATGGCTGAGCATTTCT |
