# Supplementary material for: CircMYOF triggers progression and facilitates glycolysis via the VEGFA/PI3K/AKT axis by absorbing miR-4739 in pancreatic ductal adenocarcinoma
Source: Cell Death Discov. 2021 Nov 22;7:362. doi: 10.1038/s41420-021-00759-8 (PMC8608795; doi:10.1038/s41420-021-00759-8)
Supplement: Supplementary file 3 — Supplementary Table 2 [file 41420_2021_759_MOESM3_ESM.docx]

Supplementary Table 2. Oligonucleotides sequences of sh-circMYOF

| shRNA sequences | Sequence (5’-3’) |
| --- | --- |
| sh-circMYOF-1 | GCGTAATGGCTGAGCATTTCT |
| sh-circMYOF-2 | CCGCGTAATGGCTGAGCATTT |
| sh-circMYOF-3 | CGTAATGGCTGAGCATTTCTG |
