# Supplementary material for: CircMYOF triggers progression and facilitates glycolysis via the VEGFA/PI3K/AKT axis by absorbing miR-4739 in pancreatic ductal adenocarcinoma
Source: Cell Death Discov. 2021 Nov 22;7:362. doi: 10.1038/s41420-021-00759-8 (PMC8608795; doi:10.1038/s41420-021-00759-8)
Supplement: Supplementary file 4 — Supplementary Table 3 [file 41420_2021_759_MOESM4_ESM.docx]

# Supplementary Table 3. The primer sequences used in real-time PCR.

| Gene Name | Sequence (5’-3’) |  |
| --- | --- | --- |
|  | Forward | Reverse |
| GAPDH | AGAAGGCTGGGGCTCATTTG | GCAGGAGGCATTGCTGATGAT |
| CircMYOF Divergent | cccgcgtaatggctgagca | gatcgtgtgacgcaagtcaag |
| MYOF | TCCTGCCTCTGTAGACCAAC | GAATCAATGGGGCTCGGTGA |
| U6 | CTCGCTTCGGCAGCACA | AACGCTTCACGAATTTGCGT |
| hsa-miR-1321 | GCCGAGCAGGGAGGTGAATG | GTCGTATCCAGTGCAGGGTCCGAGGTATTCGCACTGGATACGACATCACATT |
| hsa-miR-4739 | GCCGAGAAGGGAGGAGGAGC | GTCGTATCCAGTGCAGGGTCCGAGGTATTCGCACTGGATACGACAGGGCCCC |
| hsa-miR-4756-5p | GCCGAGCAGGGAGGCGCTCAC | GTCGTATCCAGTGCAGGGTCCGAGGTATTCGCACTGGATACGACAGCAGAGA |
