# Supplementary figures and images for: CircMYOF triggers progression and facilitates glycolysis via the VEGFA/PI3K/AKT axis by absorbing miR-4739 in pancreatic ductal adenocarcinoma
Source: Cell Death Discov. 2021 Nov 22;7:362. doi: 10.1038/s41420-021-00759-8 (PMC8608795; doi:10.1038/s41420-021-00759-8)

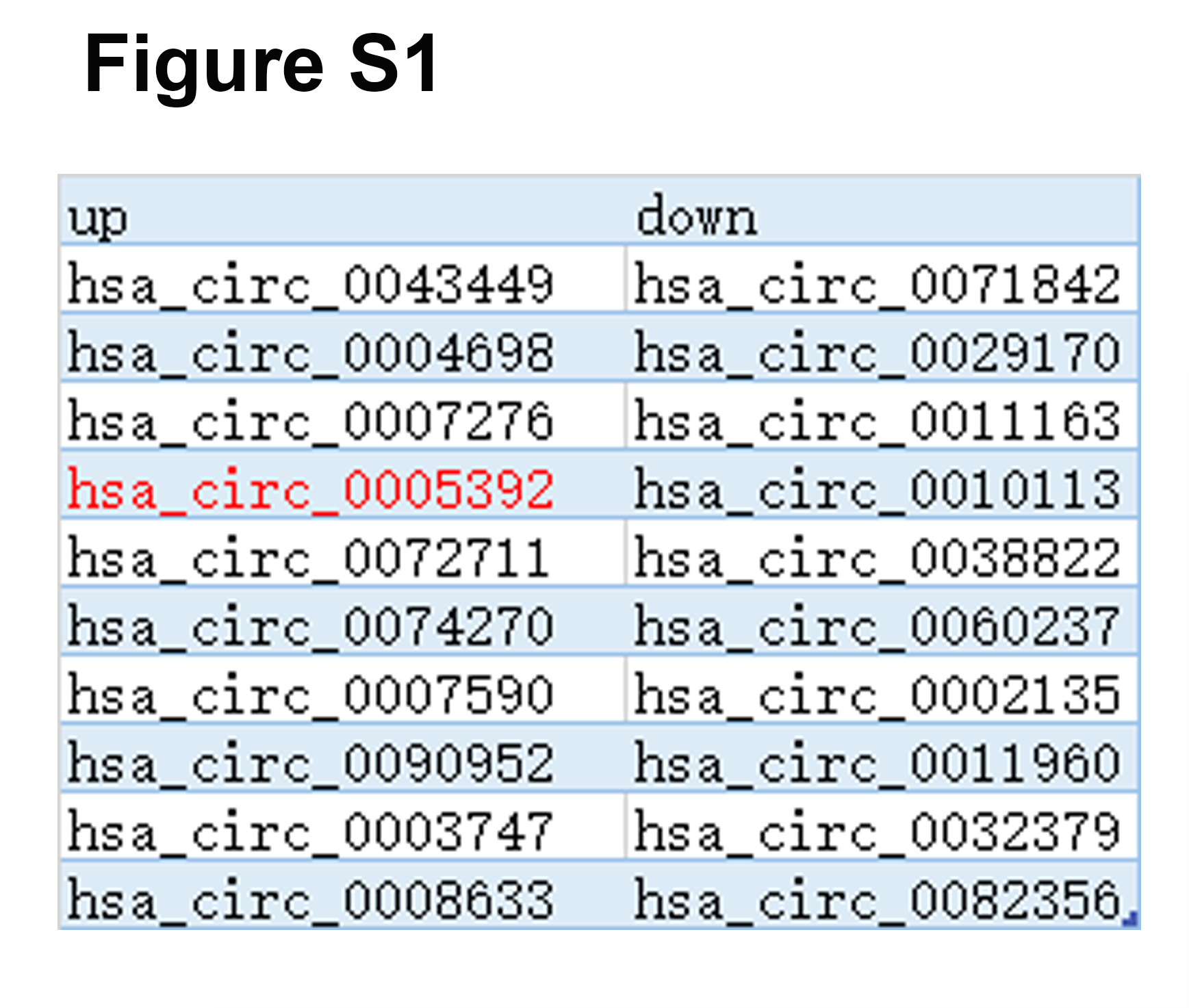

Supplement: Supplementary file 5 — Supplementary Figure 1 [file 41420_2021_759_MOESM5_ESM.tif]

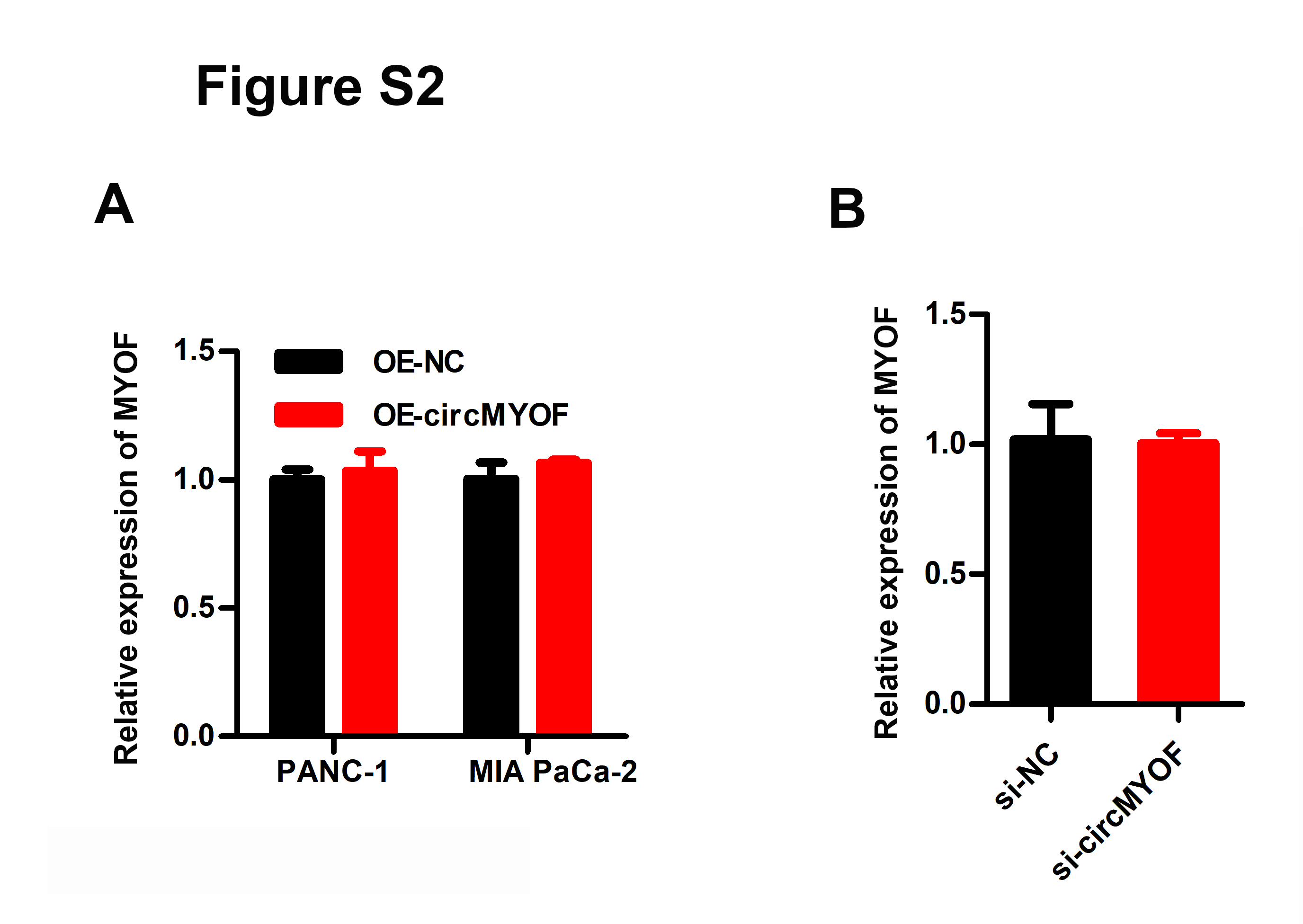

Supplement: Supplementary file 6 — Supplementary Figure 2 [file 41420_2021_759_MOESM6_ESM.tif]

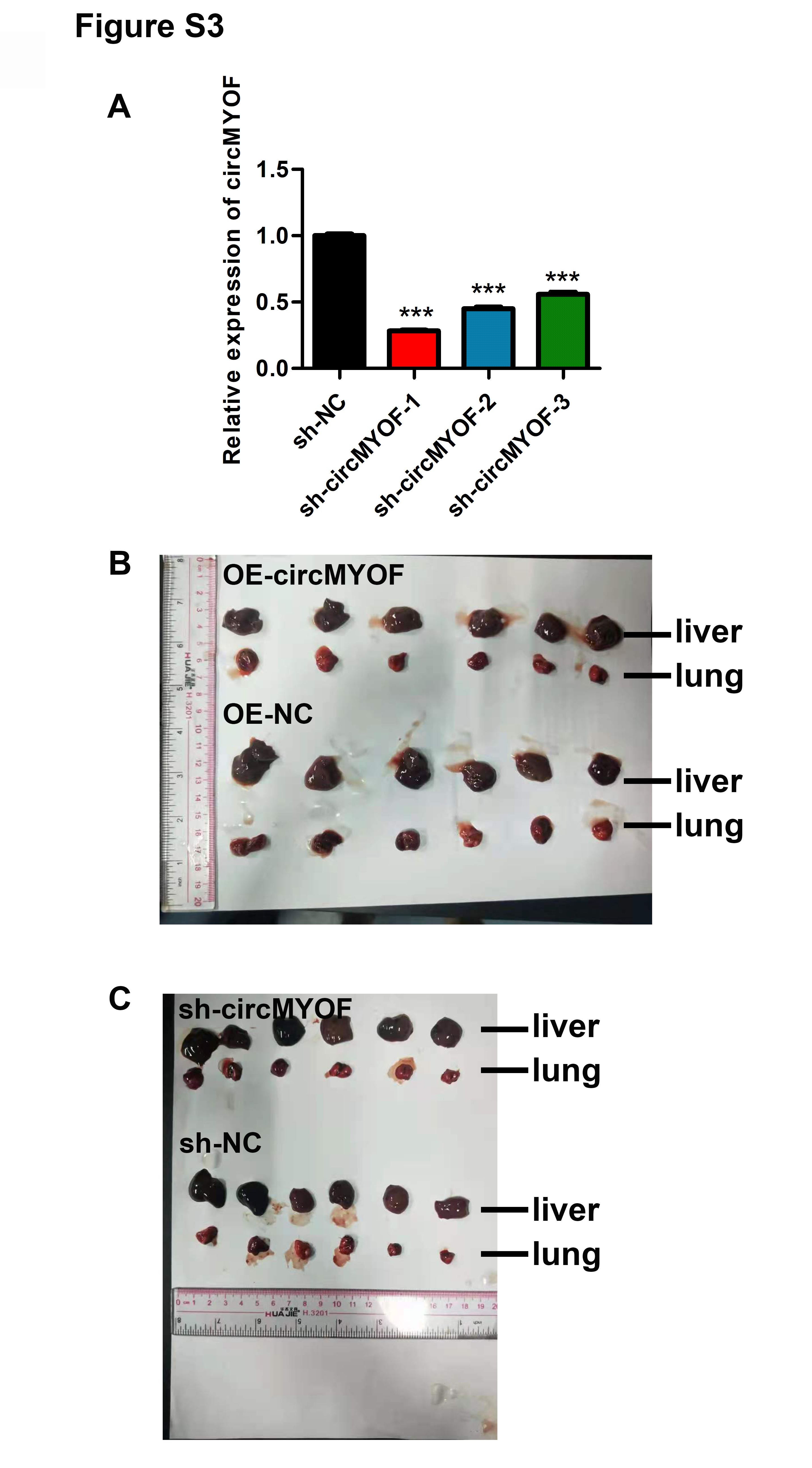

Supplement: Supplementary file 7 — Supplementary Figure 3 [file 41420_2021_759_MOESM7_ESM.tif]
