# Supplementary material for: CircMYOF triggers progression and facilitates glycolysis via the VEGFA/PI3K/AKT axis by absorbing miR-4739 in pancreatic ductal adenocarcinoma
Source: Cell Death Discov. 2021 Nov 22;7:362. doi: 10.1038/s41420-021-00759-8 (PMC8608795; doi:10.1038/s41420-021-00759-8)
Supplement: Supplementary file 8 — author contribution form [file 41420_2021_759_MOESM8_ESM.pdf]

DECLARATION OF CONTRIBUTIONS TO ARTICLE

ADMC

Manuscript Number: CDDISCOVERY-21-1999

Journal Name: *Cell Death Discovery* (the 'Journal')

Proposed Title of the Contribution:

CircMYOF triggers progression and facilitates glycolysis via VEGFA/PI3K/AKT axis by absorbing miR-4739 in pancreatic ductal adenocarcinoma (the 'Contribution)

Author(s):

Dandan Zheng, Xianxian Huang, Juanfei Peng, Yanyan Zhuang, Yuanhua Li, Junchi Qu, Shineng Zhang, Fengting Huang (the 'Authors')

For all *CDDis*covery articles, each person named as an author in the published version must be able to show he or she has contributed substantially to the article.

Authorship credit should be based on 1) substantial contributions to conception and design, acquisition of data, or analysis and interpretation of data; 2) drafting the article or revising it critically for important intellectual content; and 3) final approval of the version to be published. Authors should meet conditions 1, 2 and 3.

Any person who cannot be shown to have made a substantial contribution to the article cannot be listed as an author in the final version. The name of any person who is deemed to have made a minor contribution can, however, appear in the Acknowledgments section of the article.

Please complete the table below to indicate the contributions of all named authors to the manuscript.

Author Full Name:	Specification of Contribution to the Manuscript:
Dandan Zheng	performed development of methodology and writing the paper；provided acquisition, analysis and interpretation of data, and statistical analysis
Xianxian Huang	performed development of methodology；provided acquisition, analysis and interpretation of data, and statistical analysis
Juanfei Peng	performed development of methodology
Yanyan Zhuang	performed development of methodology
Yuanhua Li	performed development of methodology
Junchi Qu	provided acquisition, analysis and interpretation of data, and statistical analysis
Shineng Zhang	performed study concept and design；reviewed and revised of the paper
Fengting Huang	performed study concept and design；wrote and reviewed and revised of the paper

Please complete the table below to indicate the contributions of all named authors to the figures.

Figure 1:

Dandan Zheng;Xianxian Huang;Juanfei Peng;Shineng Zhang;Fengting Huang

Figure 2:

Xianxian Huang;Juanfei Peng;Yanyan Zhuang;Shineng Zhang;Fengting Huang

Figure 3:

Dandan Zheng;Xianxian Huang;Junchi Qu;Shineng Zhang;Fengting Huang

Figure 4:

Dandan Zheng;Juanfei Peng;Yanyan Zhuang;Shineng Zhang;Fengting Huang

Figure 5:

Dandan Zheng;Xianxian Huang;Yuanhua Li;Shineng Zhang;Fengting Huang

Figure 6:

Dandan Zheng;Yuanhua Li;Junchi Qu;Shineng Zhang;Fengting Huang

Signed for and on behalf of the Author(s):	Print Name:	Date:
Fengting Huang	Fengting Huang	July 1, 2021
